# Drought affects the coordination of belowground and aboveground resource‐related traits in *Solidago canadensis* in China

**DOI:** 10.1002/ece3.5536

**Published:** 2019-08-19

**Authors:** Leshan Du, Haiyan Liu, Wenbin Guan, Junmin Li, Junsheng Li

**Affiliations:** ^1^ State Environmental Protection Key Laboratory of Regional Ecological Processes and Functions Assessment Chinese Research Academy of Environmental Sciences Beijing China; ^2^ Zhejiang Provincial Key Laboratory of Evolutionary Ecology and Conservation Taizhou University Taizhou China; ^3^ Beijing Forestry University Beijing China

**Keywords:** drought stress, phenotypic plasticity, phenotypic selection, Solidago canadensis

## Abstract

Quantifying patterns of variation and coordination of plant functional traits can help to understand the mechanisms underlying both invasiveness and adaptation of plants. Little is known about the coordinated variations of performance and functional traits of different organs in invasive plants, especially in response to their adaptation to environmental stressors. To identify the responses of the invasive species *Solidago canadensis* to drought, 180 individuals were randomly collected from 15 populations and 212 ramets were replanted in a greenhouse to investigate both the response and coordination between root and leaf functional traits. Drought significantly decreased plant growth and most of the root and leaf functional traits, that is, root length, surface area, volume and leaf size, number, and mass fraction, except for the root length ratio and root mass fraction. Phenotypic plasticity was higher in root traits than in leaf traits in response to drought, and populations did not differ significantly. The plasticity of most root functional traits, that is, root length (RL), root surface area (RSA), root volume (RV), and root mass fraction (RMF), were significantly positively correlated with biomass between control and drought. However, the opposite was found for leaf functional traits, that is, specific leaf area (SLA), leaf area ratio (LAR), and leaf mass fraction (LMF). Drought enhanced the relationship between root and leaf, that is, 26 pairwise root–leaf traits were significantly correlated under drought, while only 15 pairwise root–leaf traits were significantly correlated under control conditions. Significant correlations were found between biomass and all measured functional traits except for leaf size. RV, root length ratio, RMF, total area of leaves, and LMF responded differently to water availability. These responses enable *S. canadensis* to cope with drought conditions and may help to explain the reason of the vast ecological amplitude of this species.

## 1 INTRODUCTION

Biological invasion poses a significant threat to biodiversity, and ecosystem functions both locally and globally (Mack et al., 2000; Vilà et al., 2011). The investigation of species' traits associated with invasiveness has also been a central theme in the field of invasion ecology (Moravcová, Pyšek, Jarošík, & Pergi, 2015). Plant performance traits, such as biomass, reproductive output, and plant survival contribute directly to plant fitness. However, functional traits (e.g., shoot ratio, specific leaf area, and relative reproductive allocation) can also indirectly influence plant fitness since these traits exert important influences on plant growth, survival, and reproduction (Ruprecht, Fenesi, & Nijs, 2014). Recently, several studies have quantified variations in patterns and the coordination of different plant traits, which play an important role in plant adaptations to environments (Liu et al., 2010; de la Riva, Olmo, Poorter, Ubera, & Villar, 2016). However, little is known about the coordinated variation of performance and functional traits of different organs in invasive plants, especially in response to their adaptation to environmental stressors.

Plant above‐ and belowground components are tightly linked and jointly cope with changing environmental conditions, including temperature, light, nutrient, and water availability (Craine, Froehle, Tilman, Wedin, & Chapin, 2010). Recent studies have highlighted the importance of this coordination between root and leaf functional traits in response to the limitation of above‐ and belowground resources (such as light and nutrients; Freschet, Swart, & Cornelissen, 2015; Liu et al., 2010). This coordination improves the ability of plants to acquire limiting resources or to limit their need for a particular resource (Freschet, Violle, Bourget, Scherer‐Lorenzen, & Fort, 2018). However, to date, the coordination between root and leaf traits to cope with the shortage of a single resource, especially with regard to water shortage, has not been reported in detail. Relationships between pairwise leaf–root morphology traits, such as the specific leaf area (SLA)/the specific root length (SRL), tissue density, and tissue thickness varied among different plants and in different ecosystems (Freschet, Cornelissen, Van Logtestijn, & Aerts, 2010). For example, the relationship between leaf and root tissues was previously either reported as positive (Craine et al., 2010), negative (Ryser, 1996), nonsignificant (Birouste, Kazakou, Blanchard, & Roumet, 2012; Holdaway, Richardson, Dickie, Peltzer, & Coomes, 2011), or environment‐dependent (Geng, Wang, Jin, Liu, & He, 2014). However, recent studies demonstrated that there was no unique relationship between above‐ and belowground functional traits, and different relationships were observed for arid conditions (Fort, Jouany, & Cruz, 2013; Liu et al., 2010). Thus, quantifying the patterns of variation and coordination of plant functional traits is essential and could provide an opportunity toward a detailed understanding of the mechanisms underlying invasiveness and adaptation of plants (Liu et al., 2010), particularly in the context of global climate change.

Global climate change predictions project an increase in both drought frequency and intensity as a result of anthropogenic climate change within this century (Denton, Dietrich, Smith, & Knapp, 2016; IPCC, 2013). Drought has developed into a major physiological stressor that influences the distribution, growth, physiological processes, and energy allocation of plants (Franks, 2011). For adaptation in response to drought stress, plants can modify their phenotype and biomass allocation to capture more water and reduce their water loss, thus enabling them to survive in a number of ecological niches (Alvarez‐Flores, Winkel, Nguyen‐Thi‐Truc, & Joffre, 2014). For instance, plants increase their root growth and optimize the biomass partitioning to capture more water via their roots when suffering from water shortage (Munns & Cramer, 1996). Similarly, their capacity to acquire aboveground resources is associated with the leaf area, that is, shade has been reported to increase the leaf area ratio (LAR; Ryser & Eek, 2000). Growing evidence suggests that functional trait syndromes are the outcome of adaptive strategies and their evolutionary responses, not that of single traits or unique relationships between above‐ and belowground parts (Fort et al., 2013; Morales, Squeo, Tracol, Armas, & Gutiérrez, 2015; Reich et al., 2010). However, the coordination between root and leaf traits (to cope with water shortage) has not been sufficiently studied in invasive plants.

*Solidago canadensis* L., a perennial Compositae weed native to North America, is now widely distributed throughout the world and has caused a series of ecological and economic losses in China and numerous other countries (Schittko & Wurst, 2014). In China, the species was first introduced to Shanghai in 1935 as an ornamental flower and has since then become wildly distributed in areas with high humidity, such as the coastline and the Yangtze River (Dong, Lu, Zhang, Chen, & Li, 2006; Lu et al., 2007). However, a few incidences were reported for most of the inland areas, especially in western China, which is prone to water shortage. Drought might be a key limiting factor for the distribution of *S. canadensis* and its invasion of Chinese ecosystems (Ge, He, Sun, & Chen, 2010). Understanding the patterns of phenotypic variation and response strategies to water shortage is critical to predict the invasion routes and potential distribution of *S. canadensis*.

To investigate the coordination between root and leaf traits of invasive plant species in response to drought stress, 180 individual *S. canadensis* from 15 populations in China were collected and a common garden greenhouse experiment was conducted. The plant functional traits of roots and leaves were measured, and their phenotypic plasticity was determined to address the following questions: (a) How does drought affect both root and leaf traits of *S. canadensis* from different populations? (b) Can drought affect the coordination between root and leaf traits of *S. canadensis*? (c) What is the selection direction of water availability on the root and leaf traits of *S. canadensis*?

## 2 MATERIAL AND METHODS

### 2.1 Study species and samplings

**Figure 1:**
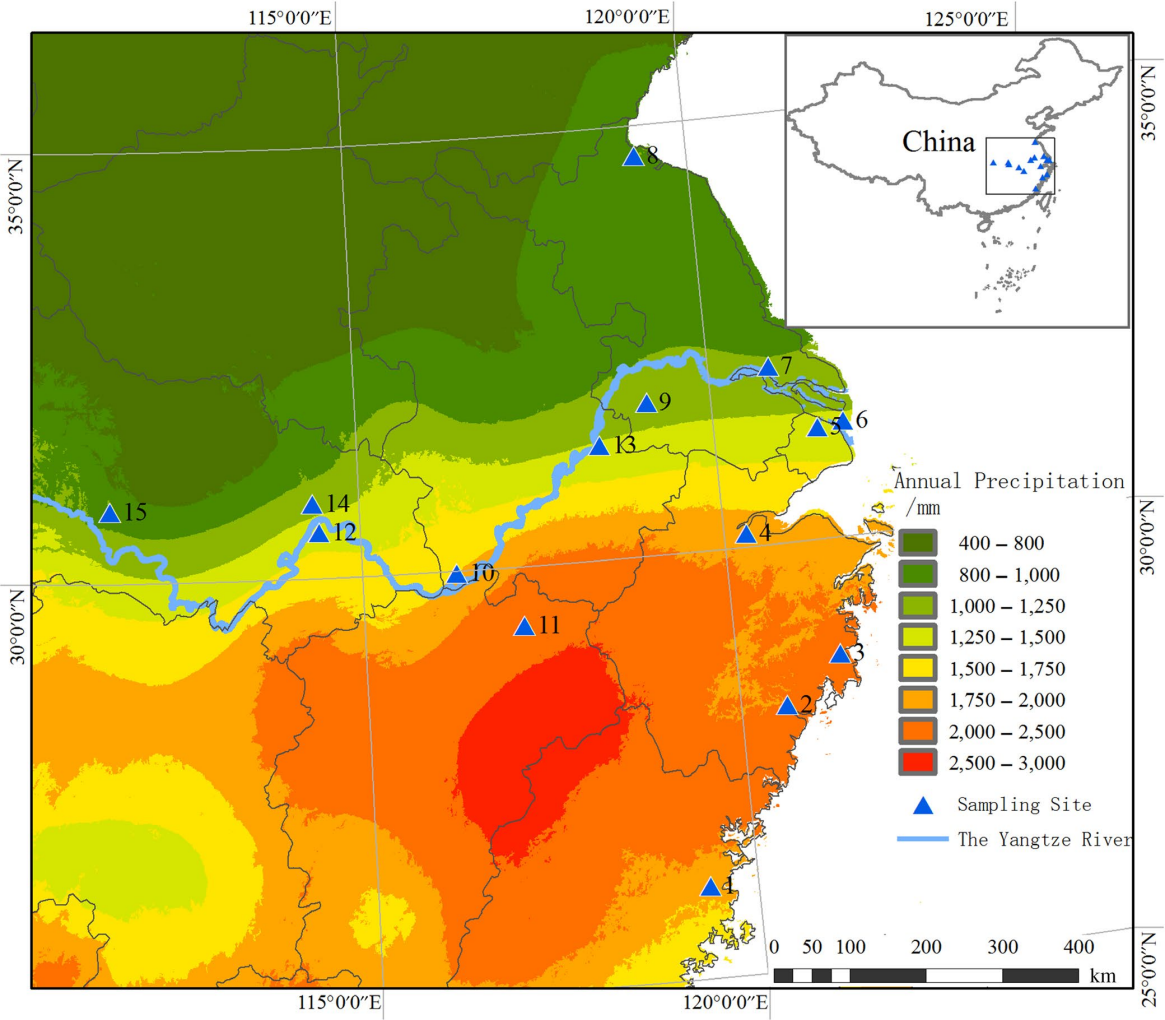
The sampling locations of *Solidago canadensis* populations in China and annual precipitation

The rhizomes of 180 *S. canadensis* individuals were collected from 15 populations in China in October 2012 (Figure 1, also see Table S1 for details about locations and environmental factors). Within each population, 12 randomly selected rhizomes were dug out, including the surrounding rhizosphere soil. These sampled rhizomes were at least 10 m from each other to avoid sampling the same clone more than once. Healthy leaves were randomly collected from 30 adult individuals in each population for genetic analysis. This was done for a total of 14 populations (the Yichang population was not used for the genetic analysis because <20 individuals were available) and 420 individuals. Leaves were immediately dried on silica gel until genetic analysis. All populations grew on abandoned farmland or vegetable gardens near roads, which represent typical invaded habitats in China.

All rhizomes were transferred and replanted into pots (diameter: 30 cm, depth: 30 cm, volume: 20 L), filled with a 3:1 mixture of sand: peat soil and were placed in a greenhouse of Taizhou University of Linhai City, Zhejiang Province, China (121°17′E, 28°87′N). The maximum water‐holding capacity of the soil mixture was measured gravimetrically (Sybilg, Jennifers, & Manuelt, 2010) as follows: Soil samples were saturated with water and then weighed. Then, the samples were dried at 50°C until they reached a constant weight (Li, Du, Guan, Yu, & Van, 2016). The genotypes of the rhizomes were identified via five microsatellite loci and were all found to be different (Li et al., 2016). Maternal effects can substantially contribute to the phenotype of an individual (And & Wulff, 1987). To avoid the maternal effect and influence of a heterogeneous environment, only newly produced ramets from the same individuals under the same conditions were used for further experimentation.

### 2.2 Genetic analysis

Total genomic DNA was extracted from dried leaves using a modified sodium dodecyl sulfate (SDS) method on FastPrep‐24 Automated Lysis and Homogenization System (MP Biomedicals). Simple sequence repeat (SSR) amplifications were performed with five primers (Table S2) in a PTC 220 Thermal Cycler (Bio‐Rad Laboratories). Due to the polyploidy of *S. canadensis*, peaks were only scored as binary data (present or absent). To detect polymorphisms in the four intron regions, PCR products were analyzed by the Fragment Analyzer™ Automated CE System (Advanced Analytical Technologies, Inc) with a capillary with 80 cm length. A DNF‐900 35–500 bp ds DNA Reagent Kit (Advanced Analytical Technologies, Inc) was used for the analysis according to the manufacturer's protocol. DNA fragments were genotyped with PROSize^®^ 2.0 Data Analysis Software according to the elution time with size standard. Nei's (1972) gene distance matrix was calculated using POPGENE ver 1.31 software (Yeh & Boyle, 1997).

### 2.3 Experimental design

Newly produced ramets of about 15 cm height were cut from the rhizomes on 6 March 2013 and were subsequently replanted in plastic pots (diameter: 16 cm, depth: 14 cm, volume: 2.8 L) using the same soil mixture that has been used for the stock population. All pots were placed on a plastic sheet to avoid water capillarity from below and were grown in a greenhouse with a transparent plastic cover to avoid the influence of precipitation.

A drought experiment was conducted in the greenhouse from 20 March 2013. Instead of plant biomass, the plant height ($H_{t_{1}}$) was recorded as the initial status at the beginning of the experiment (Du, Liu, Yan, Li, & Li, 2017). Two ramets from each of the 4–9 genotypes of the 15 *S. canadensis* population (totaling 212 ramets) were allocated to two treatments, that is, a well‐watered control and a drought treatment (per replicate, per genotype, per population, and per treatment). For the control treatment, the soil available moisture was maintained at 75%–80% of the maximum water‐holding capacity of the substrate. For the drought treatment, the soil available moisture was maintained at 20%–25%. Following previously published methods, a simple weighing method was used to appropriately adjust the pot soil moisture content (Lei, Tong, & Ding, 2006; Tian, Lu, Gong, & Shah, 2014). All pots with soil and seedlings were weighed at the beginning of the experiment and were watered every morning until they reached the weight required to maintain the target soil moisture content. No additional fertilizers were added during the whole experimental period. Pots were rearranged at random once per week to reduce potential position effects. After 2 months, all plants were harvested and relevant metrics were measured.

### 2.4 Trait measurement

Sixty days after transplanting, the total number of leaves (LN) was recorded for each plant. Plant height ($H_{t_{2}}$) and both the length and width of leaves were measured via ruler with an accuracy of 0.1 cm. The plant height increase rate was calculated as $\left(\ln\left(H_{t_{2}}\right)-\ln\left(H_{t_{1}}\right)\right)/t_{2}-t_{1}$. Because plant height does not grow at a constant rate but exponentially, plant height was Ln‐transformed before further analysis. All leaves were moved and laid on a Perspex sheet, scanned (resolution 300 dpi, Epson 1680, Seiko Epson Corporation), and their total area of leaves (LA) was measured with the computer image analysis system of WinFolia (Regent Instruments Inc). Leaf size (LS), that is, the mean area per leaf, was calculated as the ratio of the total leaf area to the number of leaves. LA represents the biomass investment, while LS is important for plant transpiration and correlates with both shade tolerance and light utilization (Freschet et al., 2015).

Furthermore, whole pots were immersed in water and then the entire root complex was excavated and carefully washed under running water to remove fine soil particles. Intact root systems were spread out in a Perspex tray (A3‐size) to minimize overlap, scanned (resolution 300 dpi, Epson 1680, Seiko Epson Corporation), and analyzed using the WinRhizo software package (Version 3.10, Regent Instruments Inc.) to obtain the total root length (RL), the root surface area (RSA), and the root volume (RV; Green, Baddeley, Cortina, & Watson, 2005). The SRL was calculated as the ratio of the total length to the root biomass. *S. canadensis* can reproduce both asexually via rhizomes and sexually via seeds. Therefore, the SRL might be biased if rhizomes were included in the analysis. However, in this study, no rhizome was found in the greenhouse treatment during the short experimental period. The root length ratio (RLR) was calculated as the ratio of the root length to the plant mass (Poorter & Ryser, 2015). Both RSA and RV indicate the water‐absorbing ability of plants. The root mass fraction (RMF) indicates the belowground biomass investment of a plant, and SRL indicates how efficient this biomass is used to increase the absorptive area. RL indicates the relative amount of water support (Markesteijn & Poorter, 2009).

Following these measurements, each plant was divided into roots, stems, and leaves at the time of harvest. Plants were then dried in an oven at 70°C until a constant weight was reached and then weighed. The SLA was calculated as the ratio of the LA to the total leaf biomass. The LAR was calculated as the ratio of the leaf area to the total biomass. The leaf mass fraction (LMF) was calculated as the ratio of the leaf mass to the total mass. The RMF was calculated as the ratio of the root mass to the total mass. LMF and RMF indicate the investment of resources to leaf and root, respectively. Both SLA and LAR indicate the light utilization efficiency of a plant (Freschet et al., 2015).

### 2.5 Statistical analysis

All traits are reported as means ± standard deviation (*SD*). Paired *t* tests were used to compare the plant height increase rate between drought treatment and control treatment. To test the influences of drought, population, and genotype, nested analysis of variance (ANOVA) using a general linear model was performed for the measured root and leaf traits. Drought treatment was used as a fixed factor, population as a random factor, and genotype as a random factor nested within population. Since populations and genotypes were randomly chosen, both population and genotype were included as random factors. All data were normally distributed and satisfied homogeneity of variance.

To explore the direction of phenotypic plasticity, a modified phenotypic plasticity index (PPI) was calculated as (*X*_drought_−*X*_control_)/*X*_drought_, where *X*_drought_ and *X*_control_ represent the mean values of drought and control treatment, respectively (Valladares, Sanchez‐Gomez, & Zavala, 2006). Plants with higher absolute PPI values have an increased advantage to cope with heterogeneous and stressful conditions. ANOVA was conducted to test the difference of PPI among populations. PPI per trait was the mean of the individual PPI traits of 15 populations (Li et al., 2016). To test whether phenotypic plasticity in response to drought contributed to an increase in biomass, a simple linear regression model (*y* = a*x* + b) was conducted between change in biomass (calculated as the biomass under drought−biomass under control) and phenotypic plasticity (Davidson, Jennions, & Nicotra, 2011).

To separately test the relationship between root and leaf functional traits under control and drought conditions, Pearson's correlations were conducted between each of the root and leaf traits. Phenotypic differences in the leaf and root resources capture ability (LA and RL), leaf and root mass fractions (LMF and RMF), leaf and root morphology (SLA and SRL), and investments in leaf and root biomass (LAR and RLR) were analyzed via linear regression to test the coordination between root and leaf traits (Freschet et al., 2015). To determine whether the slopes of the linear regression differed significantly between control and drought treatments, one‐way analysis of covariance (ANCOVA) was used to test the homogeneity of slopes (parallelism or not). The respective root trait was used as the dependent variable, treatment was used as a fixed factor, and the respective leaf trait was used as a covariant factor.

A simple linear regression model (*y* = a*x* + b) was used to predict the relationship between biomass and each measured trait separately for control and drought treatments (Du et al., 2017; Du, Yang, Guan, & Li, 2016). Here, biomass was collected as a fitness proxy since larger vegetative size is often associated with higher reproductive output (Weiner, Campbell, Pino, & Echarte, 2009). Different slopes showed the different selection directions under various tested soil moisture conditions. To determine whether the slopes of the linear regression differed significantly between control and drought treatments, ANCOVA was used to test the homogeneity of the slopes (parallelism). Biomass was used as the dependent variable, treatment was used as a fixed factor, and the respective phenotypic trait was used as the covariant factor.

All data analyses were performed with SPSS software (SPSS Inc.), and all figures were produced with Origin software (Version 9.0, OriginLab Co.).

## 3 RESULTS

### 3.1 Drought alters root and leaf functional traits

**Figure 2:**
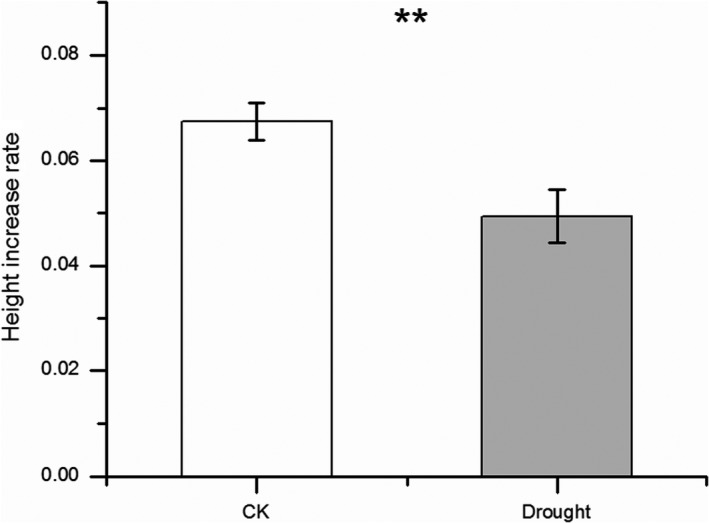
Plant height increase rate in control and drought treatments. “**” indicates a significant difference between control and drought treatment at *p* < .01. CK indicates control treatment

**Figure 3:**
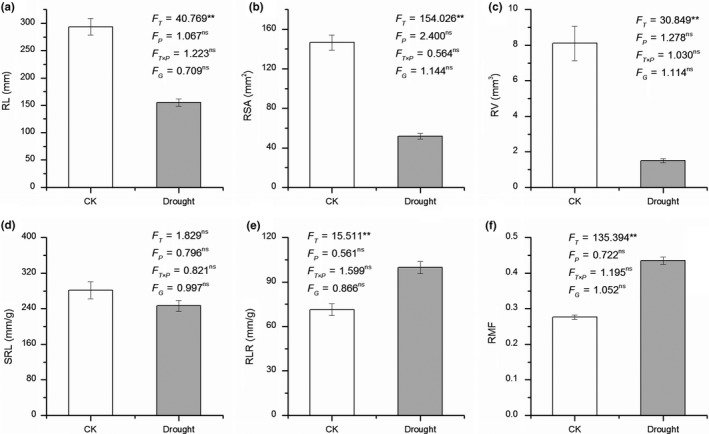
Difference of root trait between control and drought treatment. *F* value represents the result of Nested ANOVA using drought treatment as fixed factor, and both geographic population and genotype (nested within population) as random factors. *F*_T_, *F*_P_, *F*_T × P_, *F*_G_ represent the effects of drought treatment, population, the interaction between treatment and population, and genotype, respectively. “**” indicates a significant difference at *p* < .01, and “ns” indicates no significant different at *p* > .05

**Figure 4:**
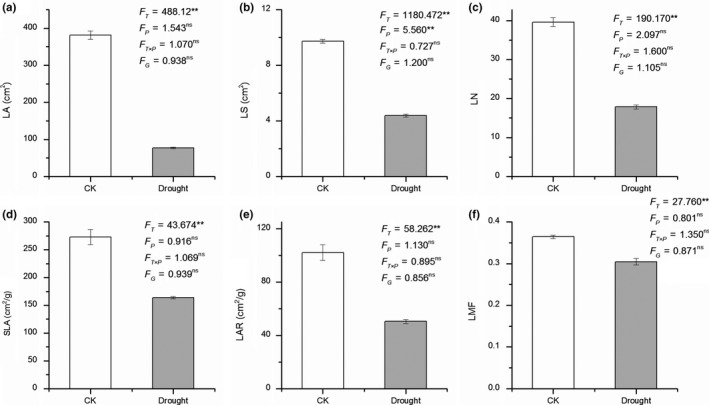
Difference of leaf traits between control and drought treatment. All figure elements are identical to those described in Figure 3

**Figure 5:**
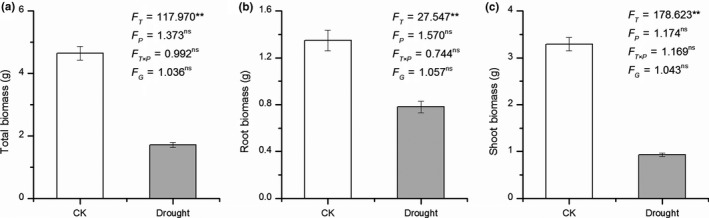
Difference of biomass between control and drought treatments. All figure elements are identical to those described in Figure 3

Drought treatment significantly decreased the rate of plant growth in height (paired *t *test = 31.243, *p* < .001, Figure 2). All root and leaf functional traits (except SRL) were significantly influenced by drought treatment (all *p* < .001 except *p* = .189 for SRL, Figure 3d), with higher RL, RSA, RV, LA, LS, LN, SLA, LAR, and LMF, but lower RLR and RMF in control than in drought treatments (Figures 3 and 4). However, population, genotype, and the interaction of population × drought exerted little effect on most of the root and leaf functional traits (*p* > .05, Figures 3 and 4). Only LS was significantly affected by populations (*p* < .05, Figure 4b). For root, shoot, and total biomass, a similar trend was found, with a significant effect for drought treatment (*p* < .05, Figure 5), and no significant effects for population, genotype, and the interaction of population × treatment (*p* > .05, Figure 5).

The phenotypic plasticity index of *S. canadensis* in response to drought treatment differed, with an average root PPI of −1.775, ranging from −6.459 (RV) to 0.323 (RMF), and an average leaf PPI of −1.637, ranging from −4.629 (LA) to −0.708 (SLA; Table 1). ANOVA showed that the difference was nonsignificant among different geographic populations. The PPI of RL, RSA, RV, and RMF were significantly positively correlated with change in biomass between drought and control treatment (*p* < .05, Table 1), while the opposite was true for the PPI of SRL, RLR, SLA, LAR, and LMF (*p* < .05, Table 1). LA, LS, and LN showed nonsignificant correlation with change in biomass (*p* > .05, Table 1).

**Table 1:** Phenotypic plasticity index (mean ± standard deviation) for root and leaf traits

Traits	Phenotypic plasticity index	*F*‐value	Linear regression coefficient between change in biomass and PPI	Correlation between distance of PPI and Nei's genetic distance
RL	−1.438 ± 0.186	1.167^ns^	0.288**	−0.02^ns^
RSA	−2.681 ± 0.268	0.75^ns^	0.439**	0.02^ns^
RV	−6.459 ± 0.778	0.614^ns^	0.354**	0.21*
SRL	−0.499 ± 0.155	0.869^ns^	−0.435**	−0.08^ns^
RLR	0.103 ± 0.092	1.335^ns^	−0.341**	0.12^ns^
RMF	0.323 ± 0.023	1.236^ns^	0.380**	0.18^ns^
LA	−4.629 ± 0.291	1.488^ns^	−0.027^ns^	−0.35***
LS	−1.332 ± 0.059	1.488^ns^	−0.189^ns^	−0.22*
LN	−1.429 ± 0.114	1.051^ns^	0.061^ns^	−0.07^ns^
SLA	−0.708 ± 0.093	1.206^ns^	−0.681**	0.14^ns^
LAR	−1.409 ± 0.206	1.142^ns^	−0.637**	0.26*
LMF	−0.318 ± 0.051	0.912^ns^	−0.452**	0.32**

Note

*F*‐value based on results of ANOVA, simple linear regression between change in biomass (biomass under drought minus control) and PPI, and correlation between distance of PPI and Nei's genetic distance.

***, **, *, and ns indicate significant correlation at *p* < 0.001, *p* < .01, *p* < .05, and *p* > .05 levels, respectively.

### 3.2 Drought enhances the coordination between root and leaf traits

When comparing pairwise root–leaf functional traits of different water availability treatments, Pearson's correlation indicated significant correlations. In the drought treatment, 26 pairwise root–leaf traits correlated significantly, of which, 16 pairwise correlations were significantly negative, and 10 pairwise root–leaf correlations were significantly positive (*p* < .05, Table 2). However, only 15 pairwise root–leaf traits significantly correlated within the control treatment, four of which showed significantly negative correlations (*p* < .05, Table 2).

**Table 2:** Pearson's correlation between leaf traits and root traits of *Solidago canadensis* treated to different water contents

Treatments	Traits	LA	LS	LN	SLA	LAR	LMF
Control	RL	0.14	−0.17	0.24**	−0.24**	−0.24**	−0.16
	RSA	0.73**	0.03	0.69**	0.08	0.04	−0.19
	RV	0.70**	0.12	0.60**	0.19	0.13	−0.15
	SRL	0.03	−0.04	0.05	0.36**	0.43**	0.56**
	RLR	0.11	0.00	0.11	0.47**	0.5**	0.38**
	RMF	0.16	0.02	0.14	−0.14	−0.25**	−0.65**
Drought	RL	0.29**	0.08	0.26**	−0.34**	−0.52**	−0.48**
	RSA	0.36**	−0.04	0.43**	−0.50**	−0.72**	−0.67**
	RV	0.33**	−0.11	0.45**	−0.52**	−0.69**	−0.64**
	SRL	−0.31**	0.12	−0.43**	0.22*	0.50**	0.51**
	RLR	−0.46**	−0.11	−0.42**	0.21*	0.18	0.14
	RMF	−0.14	−0.40**	0.15	−0.15	−0.72**	−0.81**

** and * indicate a significant correlation between control and drought treatments at *p* < .01 and *p* < .05, respectively.

**Figure 6:**
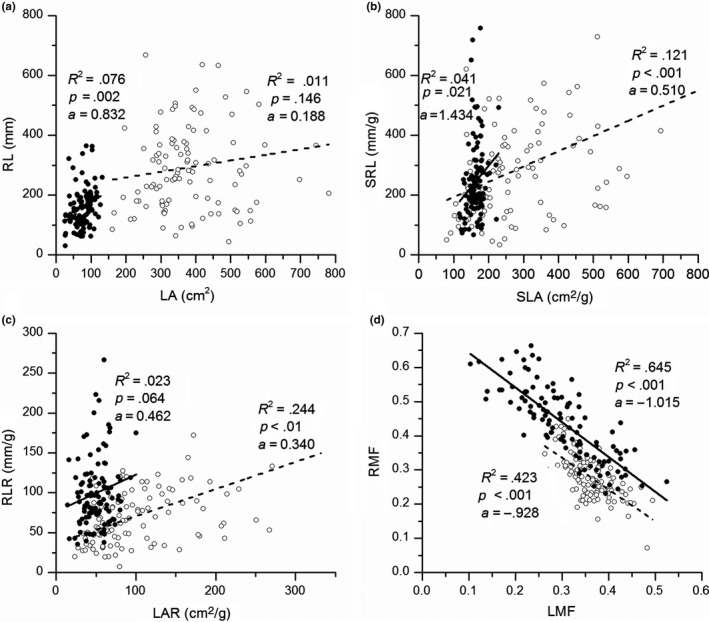
Linear regression between root length (RL) and leaf area (LA) (a), specific leaf area (SLA) and specific root length (SRL) (b), leaf area ratio (LAR) and root length ratio (RLR) (c), as well as root and leaf mass fraction (RMF and LMF, respectively) (d) in both control (empty circles) and drought treatments (solid circles). Regression analyses were separately performed for control (dashed line) and drought treatments (solid line). *R* ^2^ values represent the correlation coefficients of the regression for control and drought treatments. *p* values represent the levels of significance of the regression and *a*‐values represent regression slopes

A significant positive relationship was found between LA and RL in the drought but not in the control treatment (Figure 6a). In contrast, the relationship between LAR and RLR was not significant in drought treatment (*p* = .064), but was significant in the control treatment (Figure 6c). Significant relationships were observed between SLA and SRL, as well as between LMF and RMF, in both control and drought treatments (Figure 6b,d, all *p* < .05). Significant differences in slopes of the linear regression were observed across treatments for LA: RL, and SLA: SRL (ANCOVA, *p* < .05).

### 3.3 Selection directions of leaf and root trait responses to drought

**Figure 7:**
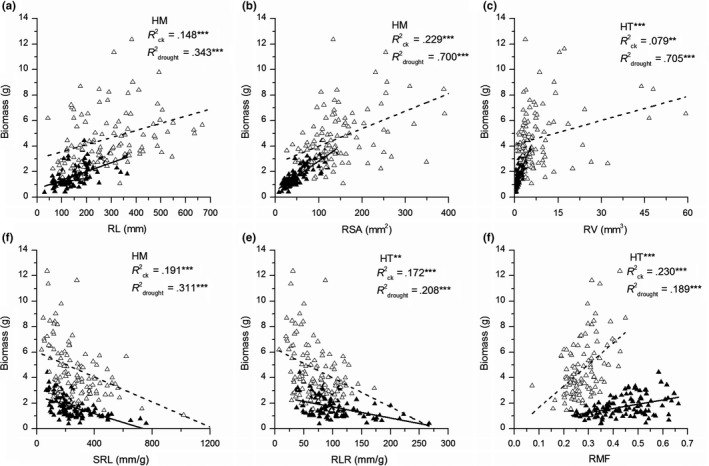
Correlations between biomass and the measured root traits of *Solidago canadensis* in both control (empty triangles) and drought treatments (solid triangles). Regression analyses were separately performed for control (dashed line) and drought treatments (solid line). $R_{\text{ck}}^{2}$ and $R_{\text{drought}}^{2}$ represent the correlation coefficients of the regression for control and drought treatments, respectively. The levels of significance of the regression are marked as **p < .01, and ***p < .001. The parallelism of the regression lines was marked as HM (parallel, *p* > .05) and HT (nonparallel, *p* < .05). “**”, and “***” indicate significant differences in the linear slope at *p* < .05, *p* < .01, and *p* < .001, respectively

**Figure 8:**
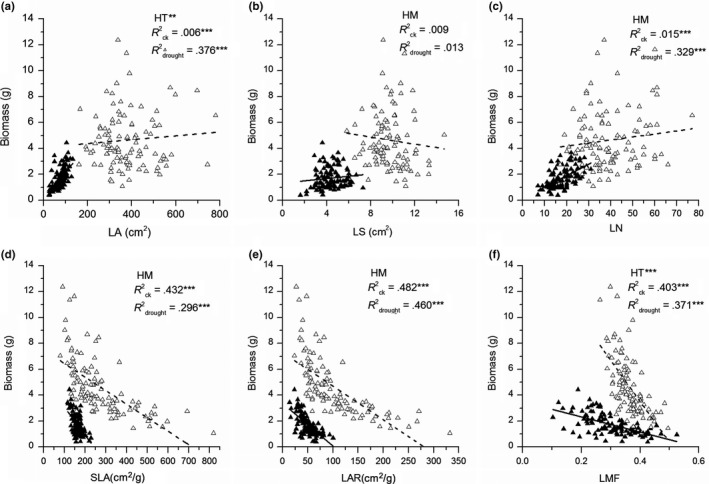
Correlations between biomass and measured leaf traits of *Solidago canadensis* in both control and drought treatments. All figure elements are identical to those described in Figure 7

In both control and drought treatments, biomass was significantly related to all root functional traits (*p* < .01, Figure 7) and leaf traits (*p* < .01, Figure 8), except for LS (*p* > .05, Figure 8b). Furthermore, the slopes of the regression lines were differed significantly in RV, RLR, and RMF (*p* < .05, Figure 7c,e,f), as well as in LA and LMF (*p* < .05, Figure 8a,f) between control and drought treatments, indicating that these traits performed differently in response to water availability.

## 4 DISCUSSION

Drought is one of the most adverse abiotic stresses for plant survival (Simova‐Stoilova et al., 2015). Severe water shortage causes detrimental effects on plants, especially on their metabolism and morphology, thus reducing growth and impeding development (Couso & Fernández, 2012; Vasellati, Oesterheld, Medan, & Loreti, 2001). In this study, drought treatment of only 20%–25% of the maximum water‐holding capacity of the soil exerted significant negative effects on the plant height growth rate, the root and leaf traits, and all biomass traits of *S. canadensis* (except SRL, RLR, and RMF). In response, resource limitations would induce plant functional adjustment consistent with an improvement of the plants' capacity to acquire the limiting resource (Freschet et al., 2018; Valladares et al., 2006; Van Kleunen & Fischer, 2005). In this study, low water availability induced high RLR and RMF, allowing a better exploration and/or exploitation of deeper soil horizons (Freschet et al., 2015), which is consistent with the root foraging theory (Alvarez‐Flores et al., 2014; Ho, Rosas, Brown, & Lynch, 2005). In contrast, SLA, LAR, and LMF decreased significantly in the drought treatment in this study, indicating a smaller leaf area per biomass (Marcelis, Heuvelink, & Goudriaan, 1998; Smedt et al., 2012). Moreover, low LA and LS could decrease water loss by reducing the amount of transpirational tissues (Markesteijn & Poorter, 2009) and thus, retain water to satisfy survival, growth, and the necessary physiological activities. Particularly, although the obtained results of biomass allocation response to drought seem to be consistent with the optimal allocation theory (Shipley & Meziane, 2002) and hypothesis of functional equilibrium (Freschet et al., 2015), this conclusion could not be substantiated based on the current results. One important reason is that nearly all of the functional traits correlated significantly with plant biomass, suggesting that the difference of partitioning is largely due to effects of drought on plant development. Moreover, unlike light and nitrogen shortage, water availability did not adjust biomass partitioning, and the optimal partitioning model did not apply to the gradient of water availability (McConnaughay & Coleman, 1999; Noulèkoun, Khamzina, Naab, & Lamers, 2017). More systematic experiments and further analyses need to be conducted.

Nested ANOVA using a general linear model showed that drought significantly affected plant functional traits, while population, genotype, and the interaction of population × drought exerted little effects on most root and leaf functional traits, with the exception that LS was significantly affected by populations. A similar result was found for PPI, where all mean PPI of traits were not significantly different among geographic populations. The results suggest that the leaf and root traits of *S. canadensis* were genetically stable. The lower variation of traits among populations of *S. canadensis* might be due to the short invasion history and weak selection pressure in different populations of this species (Li, Liu, Yan, & Du, 2017). In addition, the large amounts of gene flow among populations, with extensive pollen and seed movements of *S. canadensis* (Moran, Reid, & Levine, 2017), might be another reason.

Phenotypic plasticity for a given trait may exert important adaptive effects, thus minimizing adverse environmental effects while maximizing survival, growth, and reproduction (Valladares & Niinemets, 2008). Variation of the plasticity of ecologically important traits for the same species across various environments was determined by different strategies and selective pressures (Couso & Fernández, 2012). In this study, the phenotypic plasticity index of *S. canadensis* in response to drought treatment differed, with an average root PPI of −1.775, ranging from −6.459 (RV) to 0.323 (RMF), and an average leaf PPI of −1.637, ranging from −4.629 (LA) to −0.708 (SLA). This variation of plasticity may lower the cost of phenotypic plasticity (Sergei, 2010) and help the plant to maintain adaptation across a range of environments, which further influences their distribution and ecological breadth. Moreover, change in biomass between drought and control treatments correlated positively with most PPI of root traits (RL, RSA, RV, and RMF), but correlated negatively with most PPI of leaf traits (SLA, LAR, and LMF), indicating that increased root plasticity and decreased leaf plasticity will induce more biomass in response to water shortage. In light of these results, phenotypic plasticity can be assumed to play an important role for the response of *S. canadensis* to drought, and potentially contributes to an expansion of ecological niches, which is a further indication why *S. canadensis* is wide‐spread throughout China and other countries across the world (Schittko & Wurst, 2014).

Plant above‐ and belowground components are tightly linked (Geng et al., 2014). Plants with a closer correlation between roots and leaves likely grow and survive more successfully when they are exposed to resource limitations (Freschet et al., 2015). This was indicated by the current study, where among 36 pairwise root–leaf traits of the invasive *S. canadensis* in the control treatment, 15 pairwise root–leaf traits coordinations were detected. In contrast, among 36 pairwise root–leaf traits of the invasive *S. canadensis* in drought treatment, 26 pairwise root–leaf traits coordinations were detected. For example, LMF showed no significant relationship with root traits (RL, RSA, and RV) under the control treatment, while it exhibited a significant correlation under drought treatment. Besides, drought treatment significantly enhanced the coordination of the relationship between LA and RL, as well as SLA:SRL (ANCOVA, *p* < .05), which was related to root and leaf morphologies and the resource capture ability of plants. The relationship between above‐ and belowground traits was strengthened in combination with the resource shortage. In summary, drought enhanced the leaf–root trait coordination, and this coordinated performance was essential for the faster resource acquisition of *S. canadensis* in response to water shortage (Freschet et al., 2015; Laughlin, Leppert, Moore, & Sieg, 2010).

Linear regression analysis showed that the relationship between biomass and both the root and leaf functional traits of *S. canadensis* under control and drought treatments were significant, except for LS. In addition, the selection direction of water availability on LMF, LA, RV, RLR, and RMF differed, indicating that the selection differentials of water availability on these traits were significant under different soil moisture conditions (Bo, Shibuya, Yogo, Hara, & Yokozawa, 2001; Du et al., 2016). This study also found significant relationships between genetic differentiation and phenotypic differentiation based on PPI of LMF, LA, RV, LAR, and LS at the population level, indicating that the combination effect of selection and genetic drift might differ (Abbott, DuBois, Grosberg, Williams, & Stachowicz, 2018). For LMF, LA, and RV, the effect of selection might be the main direction, but for RLR, RMF, LAR, and LS, the effect of selection might be neutralized by genetic drift. Environmental differences across a population create varying selection pressures that drive the differentiation of traits, while limiting gene flow within the population and allowing divergence of neutral genetic markers (Abbott et al., 2018).

In conclusion, the presented results demonstrate that drought exerted significant adverse effects on the root and leaf traits of *S. canadensis*. In response, *S. canadensis* showed high phenotypic plasticity and invested more resources into root organs to capture more water and nutrients. Besides, enhanced correlations between root and leaf trait were driven by drought, which might be utilized in resource use strategies. Both environmental factor and genetic drift affected the root and leaf traits of *S. canadensis* at the population level. Further studies should test the response of *S. canadensis* to other environmental stressors to identify general rules of the coordination between root and leaf traits and to investigate the molecular mechanism underlying this coordination.

## CONFLICT OF INTEREST

None declared.

## AUTHOR CONTRIBUTIONS

LJM, LJS, DL, and GW conceived the study. DL and LH conducted the field works. LJM, DL, and LH conducted the analyses. LJM, DL, and LH wrote the manuscript. All authors read, revised, and approved the manuscript.

## Data Availability

We agree to make our data publicly available in a relevant repository. Sampling locations and morphological data: Dryad https://doi.org/10.5061/dryad.cr80m54
