# Supplementary material for: Drought affects the coordination of belowground and aboveground resource‐related traits in Solidago canadensis in China
Source: Ecol Evol. 2019 Aug 19;9(17):9948–60. doi: 10.1002/ece3.5536 (PMC6745655; doi:10.1002/ece3.5536)
Supplement: Supplementary file 1 [file ECE3-9-9948-s001.docx]

# Table S1. Locations and environmental factors of the fifteen populations in this study

| No. | Population | Location | Longitude | Latitude | Altitude（m） | Annual precipitation (mm) | Annual mean temperature (℃) | Habitats |
| --- | --- | --- | --- | --- | --- | --- | --- | --- |
| 1 | FZ | Fuzhou City, Fujian Province | N119.359º | E26.098º | 19 | 1375 | 20.1 | Abandoned Farmland |
| 2 | WZ | Wenzhou City, Zhejiang Province | N120.607º | E28.126º | 4 | 1356 | 16.0 | Abandoned Farmland |
| 3 | TZ | Taizhou City, Zhejiang Province | N121.397º | E28.656º | 6 | 1717 | 17.5 | Abandoned Farmland |
| 4 | HZ | Xiaoshan District, Hangzhou City, Zhejiang Province | N120.297º | E30.161º | 9 | 1075 | 16.9 | Abandoned farmland |
| 5 | HQ | Minhang District, Shanghai City | N121.433º | E31.307º | 5 | 1571 | 16.2 | Green belts |
| 6 | PD | Pudong District, Shanghai City | N121.804º | E31.354º | 3 | 1175 | 15.9 | Abandoned farmland |
| 7 | NT | Nantong City, Jiangsu Province | N120.843º | E32.070º | 5 | 1123 | 15.0 | Abandoned farmland |
| 8 | LYG | Lianyungang City, Jiangsu Province | N19.235º | E34.654º | 3 | 1190 | 13.5 | Abandoned farmland |
| 9 | NJ | Nanjing City, Jiangsu Province | N119.094º | E31.794º | 22 | 1252 | 15.7 | Abandoned farmland |
| 10 | JJ | Jiujiang City, Jiangxi Province | N116.283º | E29.985º | 18 | 864 | 17.2 | Abandoned vegetable garden |
| 11 | JDZ | Jingdezhen City, Jiangxi Province | N117.166º | E29.318º | 40 | 1420 | 17.7 | Green belts |
| 12 | WC | Wuchang District, Wuhan City, Hubei Province | N114.421º | E30.541º | 26 | 1043 | 17.3 | Abandoned vegetable garden |
| 13 | WHu | Wuhu City, Anhui Province | N118.387º | E31.342º | 16 | 1774 | 16.3 | Garbage dump |
| 14 | WH | Hankou District, Wuhan City, Hubei Province | N114.350º | E30.878º | 25 | 1022 | 17.1 | Abandoned farmland |
| 15 | YC | Yichang City, Hubei Province | N111.532º | E30.843º | 333 | 1068 | 15.7 | Abandoned building land |

# Table S2. Sequences of 4 primers used in SSR analysis of *Solidago canadensis L.*

| Primer | Sequences（5’--3’） | Fragment size |
| --- | --- | --- |
| SS1B | F: TTCCTGAAGAAGCTTCGCATA  R: CAGCAGATGCATTCCATAA | 156-210 |
| SS4F | F: ACACGTGGACCAGGTAAAGC  R: CGCGAAGAACAGCAATACAA | 168-192 |
| SS20E | F: CACACAGACACTCAAAGCTTCA  R: ACCCGCCCTAAAAATAAAGA | 273-299 |
| SS24F | F: AGVTTTTCTTCGCCATTTCCTTCC  R: AATTTGGTTACTGGGTTTTCTTGA | 156-222 |
| SS4G | F: TGTGACAGCTTGTTAACTTTATACTGA  R: CACCCCCTTTCCAAATATGA | 171-227 |

# Table S3. Nei’s genetic distances based on SSR data

| Population | FZ | HZ | JD | JJ | LY | NJ | NT | SH | SH | TZ | WZ | WHU | WC |
| --- | --- | --- | --- | --- | --- | --- | --- | --- | --- | --- | --- | --- | --- |
| HZ | 0.010 |  |  |  |  |  |  |  |  |  |  |  |  |
| JD | 0.011 | 0.010 |  |  |  |  |  |  |  |  |  |  |  |
| JJ | 0.012 | 0.015 | 0.005 |  |  |  |  |  |  |  |  |  |  |
| LY | 0.008 | 0.011 | 0.008 | 0.009 |  |  |  |  |  |  |  |  |  |
| NJ | 0.013 | 0.010 | 0.006 | 0.007 | 0.008 |  |  |  |  |  |  |  |  |
| NT | 0.010 | 0.012 | 0.010 | 0.009 | 0.005 | 0.008 |  |  |  |  |  |  |  |
| SH | 0.013 | 0.010 | 0.014 | 0.016 | 0.012 | 0.012 | 0.016 |  |  |  |  |  |  |
| SH | 0.008 | 0.010 | 0.006 | 0.004 | 0.007 | 0.007 | 0.007 | 0.012 |  |  |  |  |  |
| TZ | 0.014 | 0.009 | 0.012 | 0.014 | 0.016 | 0.012 | 0.015 | 0.011 | 0.011 |  |  |  |  |
| WZ | 0.012 | 0.011 | 0.011 | 0.016 | 0.013 | 0.013 | 0.017 | 0.012 | 0.012 | 0.014 |  |  |  |
| WHU | 0.021 | 0.015 | 0.009 | 0.013 | 0.017 | 0.012 | 0.018 | 0.018 | 0.012 | 0.013 | 0.015 |  |  |
| WC | 0.014 | 0.013 | 0.008 | 0.011 | 0.011 | 0.010 | 0.013 | 0.015 | 0.008 | 0.011 | 0.014 | 0.011 |  |
| WH | 0.013 | 0.010 | 0.009 | 0.012 | 0.016 | 0.014 | 0.018 | 0.016 | 0.010 | 0.010 | 0.016 | 0.012 | 0.013 |
